# Supplementary material for: Metallic porous nitride single crystals at two-centimeter scale delivering enhanced pseudocapacitance
Source: Nat Commun. 2019 Oct 17;10:4727. doi: 10.1038/s41467-019-12818-x (PMC6797774; doi:10.1038/s41467-019-12818-x)
Supplement: Supplementary file 1 — Supplementary Information [file 41467_2019_12818_MOESM1_ESM.pdf]

Metallic porous nitride single crystals at 2 cm scale delivering enhanced pseudocapacitance

Xi et al.

Supplementary Figures

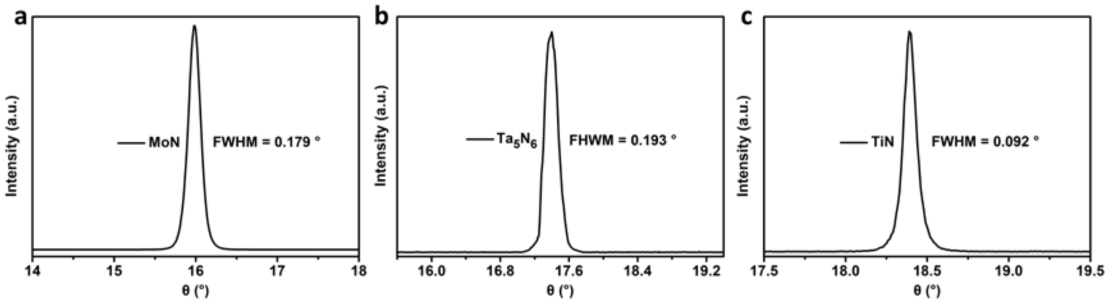

Supplementary Fig.1. **The rocking curve curves of the porous single crystals.** The FWHM is 0.179, 0.193 and 0.092° for the porous (**a**) MoN, (**b**) Ta_5N_6 and (**c**) TiN single crystals, respectively.

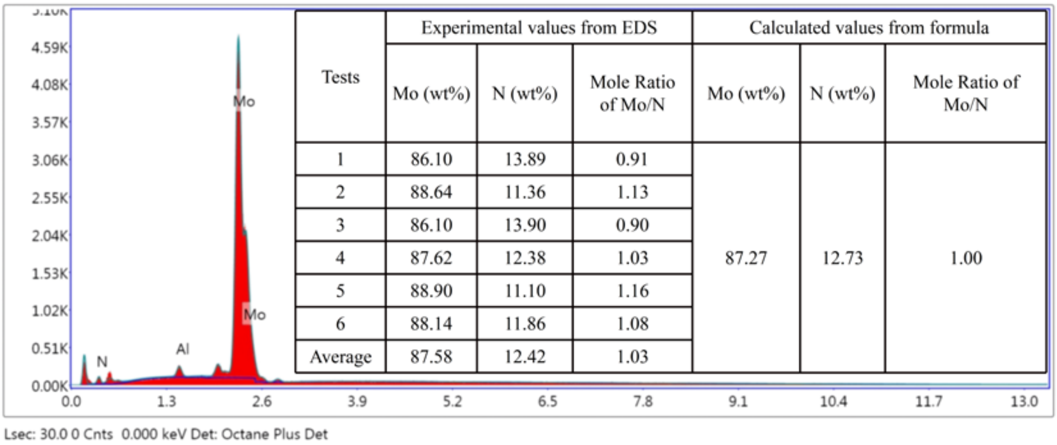

Tests	Experimental values from EDS			Calculated values from formula		
	Mo (wt%)	N (wt%)	Mole Ratio of Mo/N	Mo (wt%)	N (wt%)	Mole Ratio of Mo/N
1	86.10	13.89	0.91	87.27	12.73	1.00
2	88.64	11.36	1.13			
3	86.10	13.90	0.90			
4	87.62	12.38	1.03			
5	88.90	11.10	1.16			
6	88.14	11.86	1.08			
Average	87.58	12.42	1.03			

Supplementary Fig.2. **The element analysis of MoN single crystal.** No oxygen residual is observed. The mole ratio between N and Mo is approximately at 1.0.

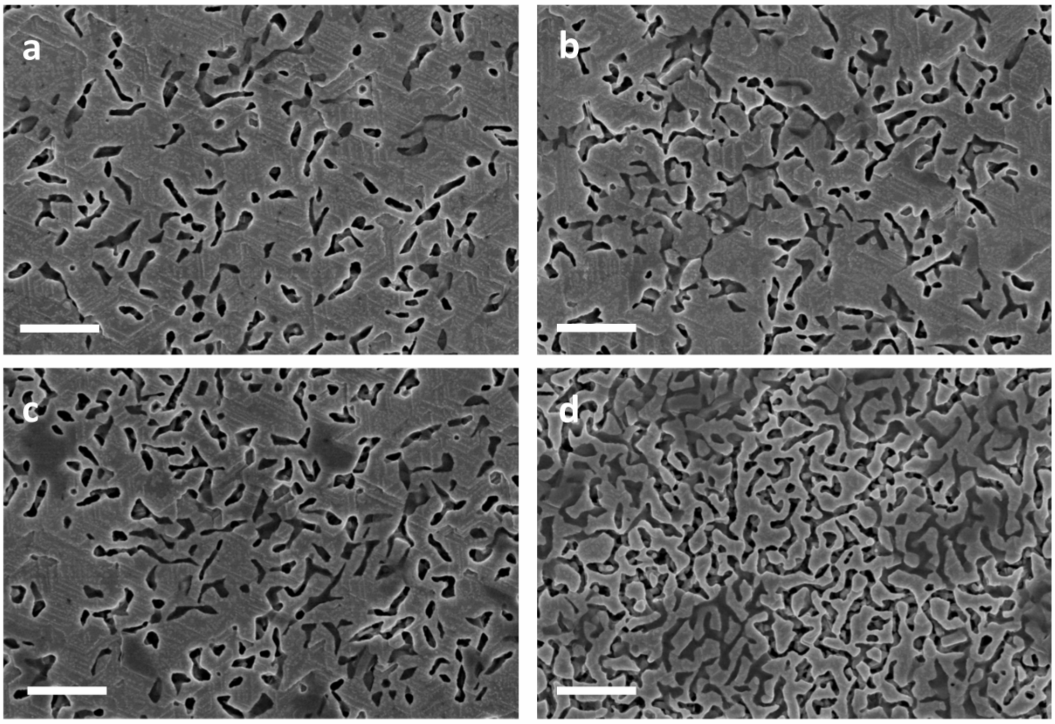

Supplementary Fig.3. The porous microstructures of MoN single crystals. They are grown with lattice reconstruction strategy from $PbMoO_4$ parent crystals under different ammonia pressures. (**a**) 500 Torr. (**b**) 300 Torr. (**c**) 100 Torr and (**d**) 50 Torr. The scale bar is 1 μm in (**a**), (**b**), (**c**) and (**d**).

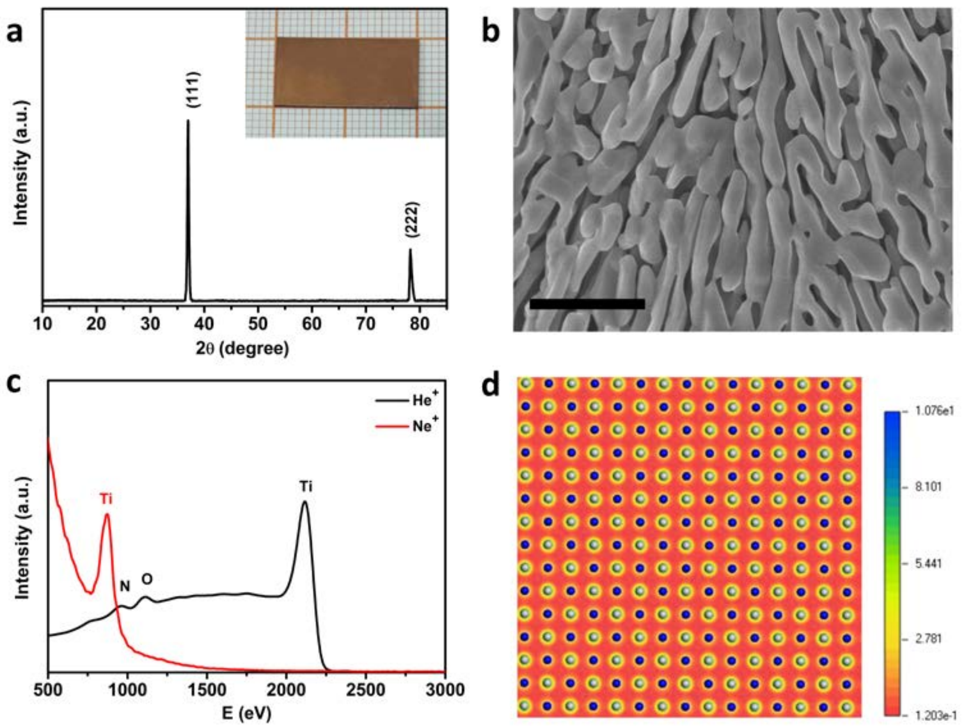

Supplementary Fig.4. Crystal structure and microstructure of porous TiN single crystal. (**a**) The XRD pattern of porous TiN single crystal. Inset image shows optical photograph of porous TiN single crystal with dimensions of 20 mm × 10 mm × 0.5 mm. (**b**) The SEM image of porous TiN single crystal. (**c**) HS-LEIS spectra of the outmost surface layer of porous TiN single crystal. (**d**) The simulative charge density graph on the surface of porous TiN crystal; gold and blue ball stand for Ti and N element, respectively. The scale bar is 500 nm in (**b**).

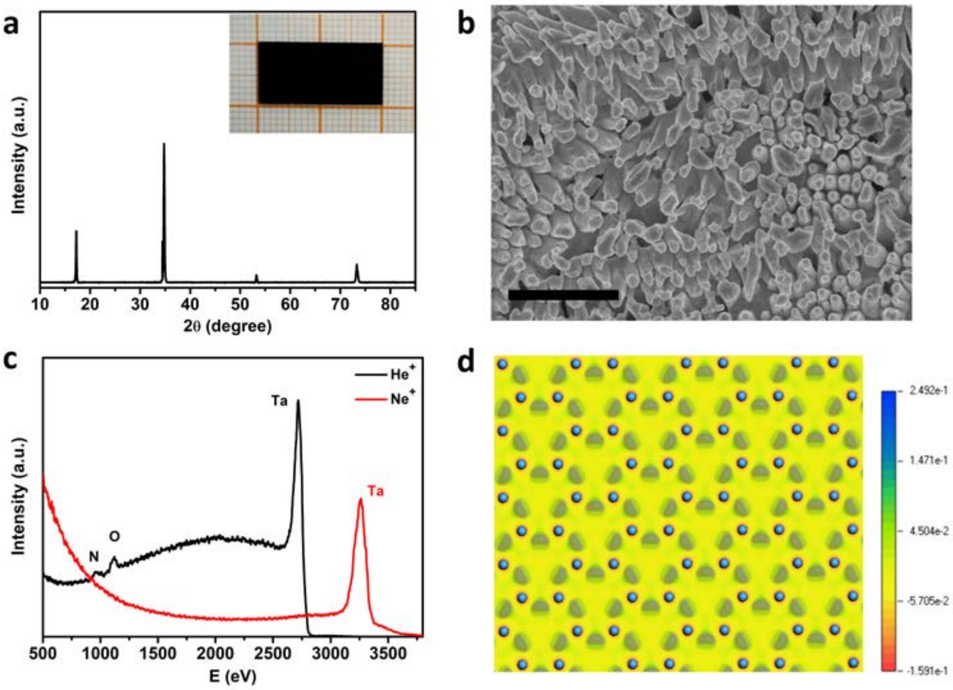

Supplementary Fig.5. Crystal structure and microstructure of porous Ta_5N_6 single crystal. (**a**) The XRD pattern of porous Ta_5N_6 single crystal. Inset image shows optical photograph of porous Ta_5N_6 single crystal with dimensions of 20 mm × 10 mm × 0.5 mm. (**b**) The SEM image of porous Ta_5N_6 single crystal. (**c**) HS-LEIS spectra of the outmost surface layer of porous Ta_5N_6 single crystal. (**d**) The simulative charge density graph on the surface of porous Ta_5N_6 crystal; grey ball stand for Ta element. The scale bar is 500 nm in (**b**).

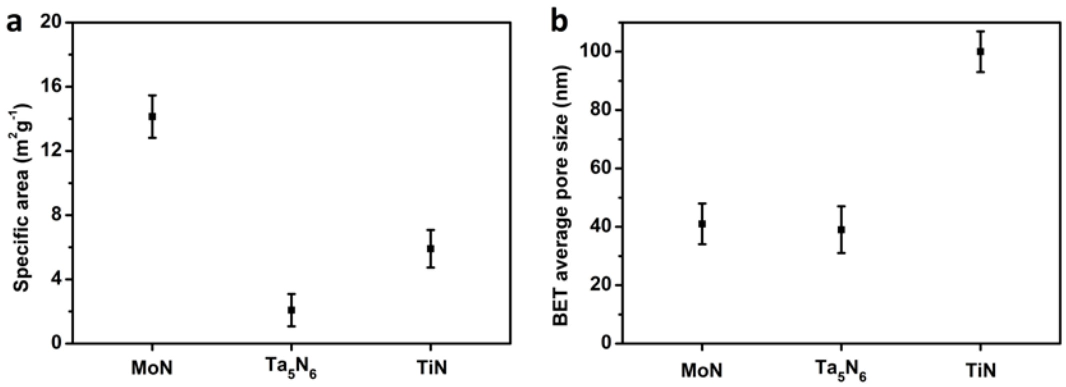

Supplementary Fig.6. Specific area and pore size of porous single crystals. The specific surface area (**a**) and average pore size (**b**) of the three porous nitride single crystals. The error bars represent standard deviation in repeated measurements.

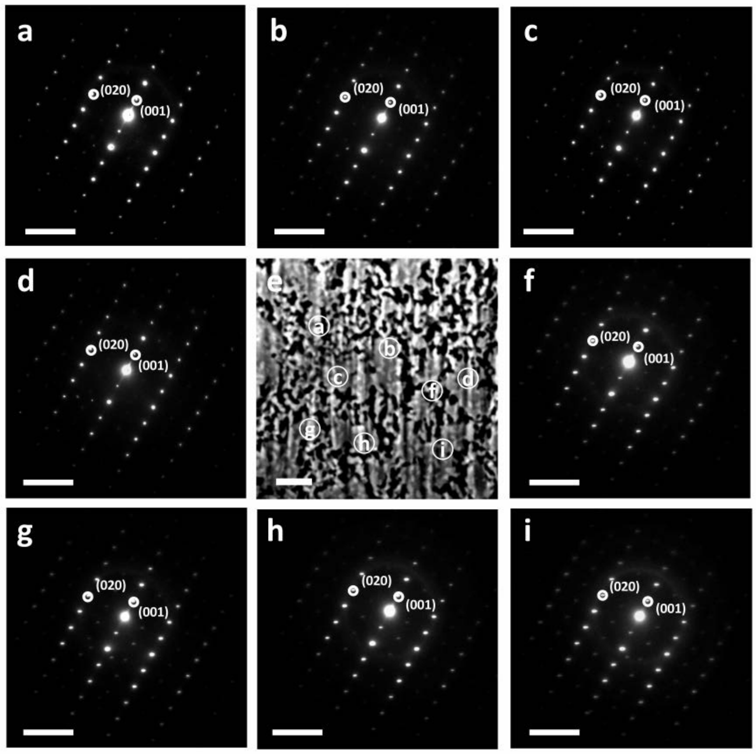

Supplementary Fig.7. Cross-sectional view and selected area electron diffraction (SAED). The porous single-crystalline MoN is grown on the [001] $PbMoO_4$ substrate. The (**a**)-(**d**) and (**f**)-(**i**) present the SAED pattern at different locations on the cross-section of the porous single-crystalline MoN. The (**e**) represents the cross-sectional view of the porous single-crystalline MoN with the locations for SAED patterns labeled. The scale bar is 5 1/nm in (**a**)-(**d**) and (**f**)-(**i**). The scale bar is 1 μm in (**e**).

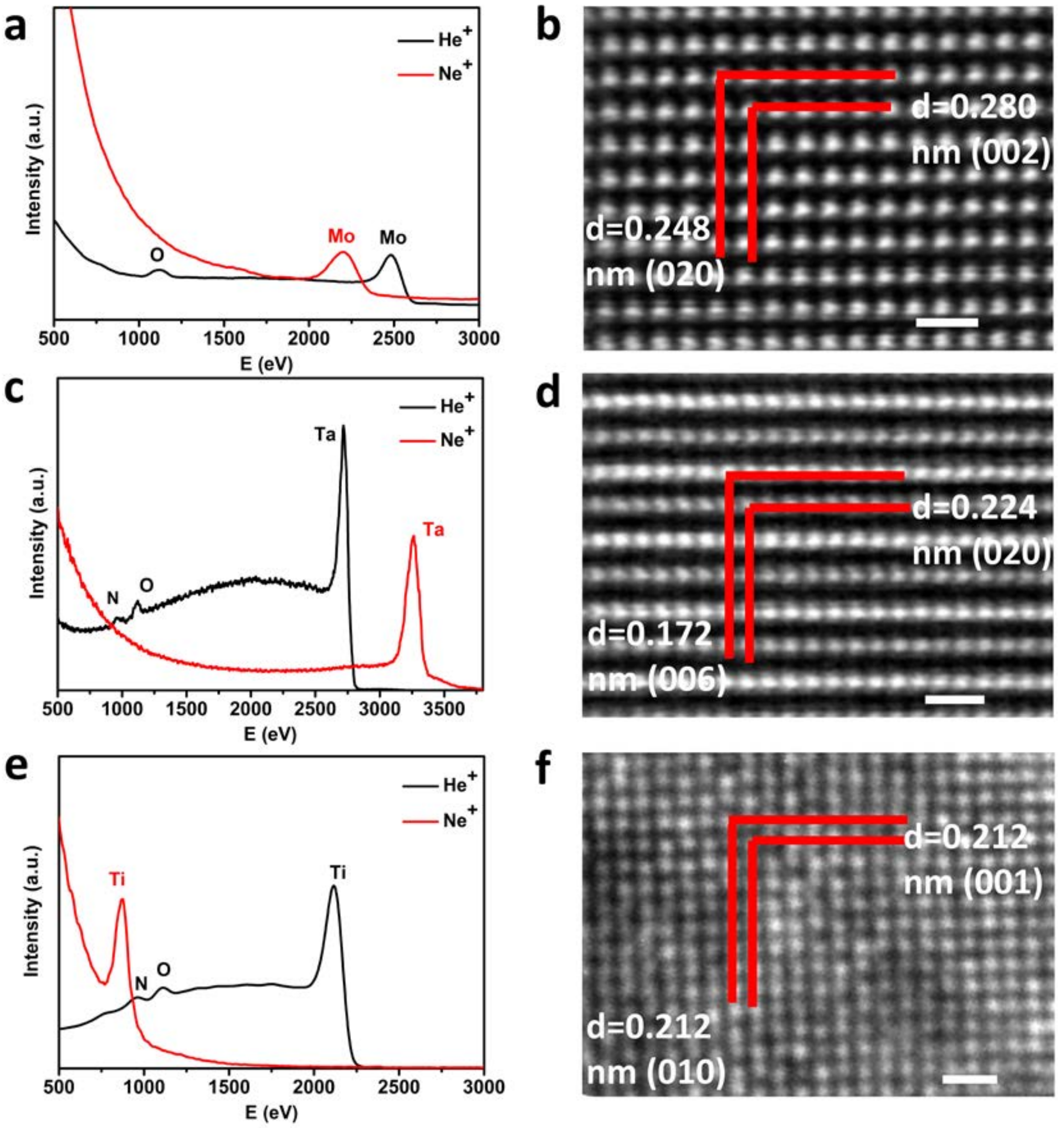

Supplementary Fig.8. **Surface termination layer and crystal structure of porous cyrstals.** The high-sensitive low energy ion scattering (HS-LEIS) with He^+ (3 keV) and Ne^+ (5 keV) ions for porous (**a**) MoN, (**c**) TiN and (**e**) Ta_5N_6 single crystals. The high-resolution Cs-corrected STEM pictures of porous (**b**) MoN, (**d**) TiN and (**f**) Ta_5N_6 single crystals. The scale bar is 1 nm in (**b**), (**d**) and (**f**).

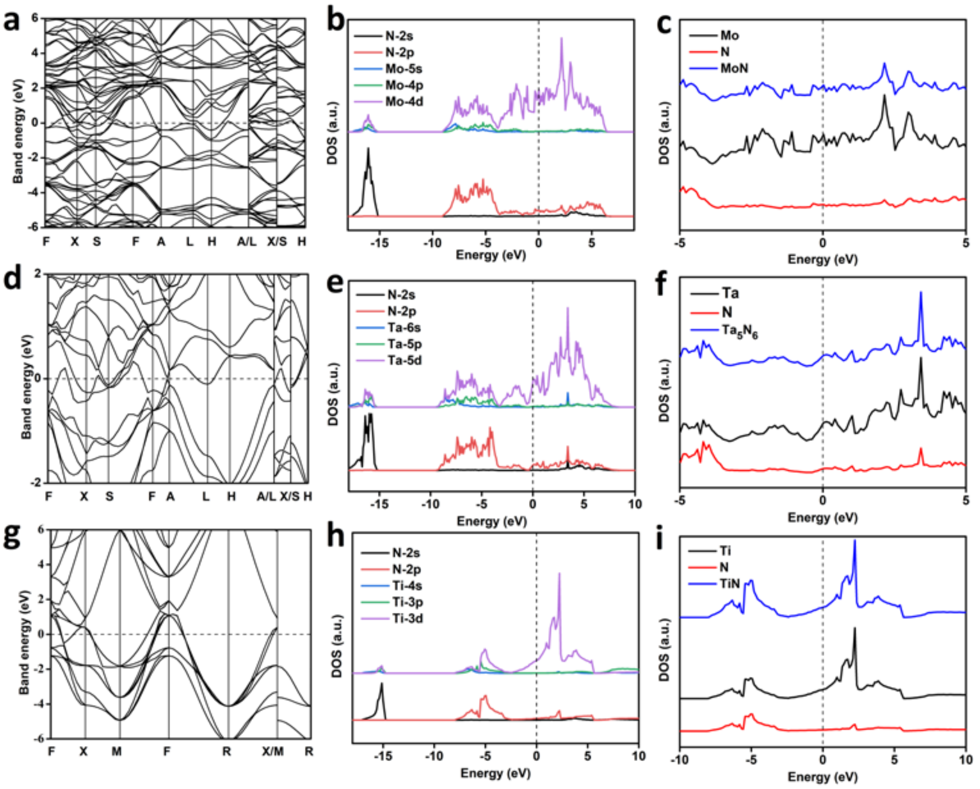

Supplementary Fig. 9. The band gaps and density of states of the three different nitrides. (**a-c**) The band gaps and electronic structures of MoN. (**d-f**) The band gaps and electronic structures of Ta_5N_6. (**g-i**) The band gaps and electronic structures of TiN.

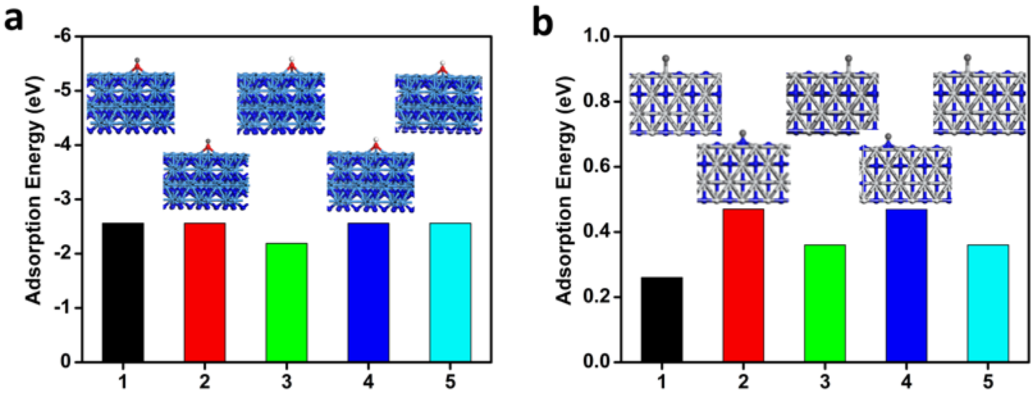

Supplementary Fig10. Chemisorption energy of TiN single crystal. (**a**) The calculated adsorption energy of OH^- adsorption on Ta_5N_6 surface. (**b**) The H^+ adsorption on TiN surface. Inset images are the corresponding adsorption configurations.

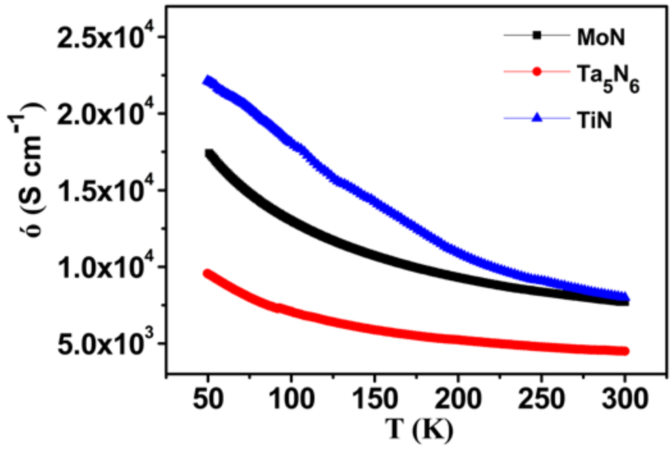

Supplementary Fig.11. **Electrical conductivity of the crystals.** The relationship between conductivity and temperature for the porous MoN, Ta_5N_6 and TiN single crystals. The porous single crystals demonstrate metallic conduction behaviors.

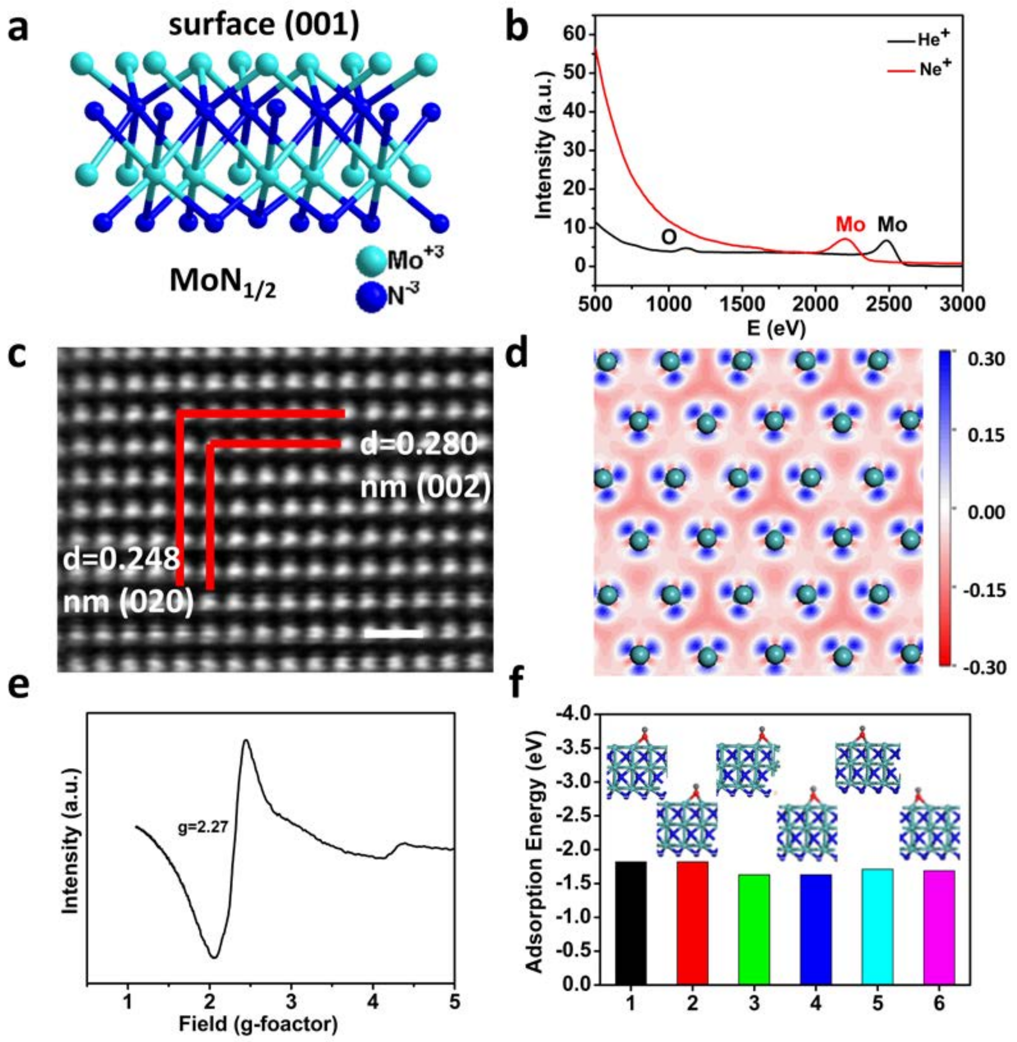

Supplementary Fig.12. **The coordination structures for the porous MoN single crystal.** (**a**) The surface structure model of MoN with <001> orientation. (**b**) The high-sensitive low energy ion scattering (HS-LEIS) with He^+ (3 keV) and Ne^+ (5 keV) ions for porous MoN crystal. (**c**) The STEM picture of MoN single crystal. (**d**) The simulative charge density graph on the surface of porous MoN crystal; green ball stands for Mo element. (**e**) The ESR of MoN single crystal. (**f**) The chemisorption configurations on MoN surface. The scale bar is 1 nm in (**c**).

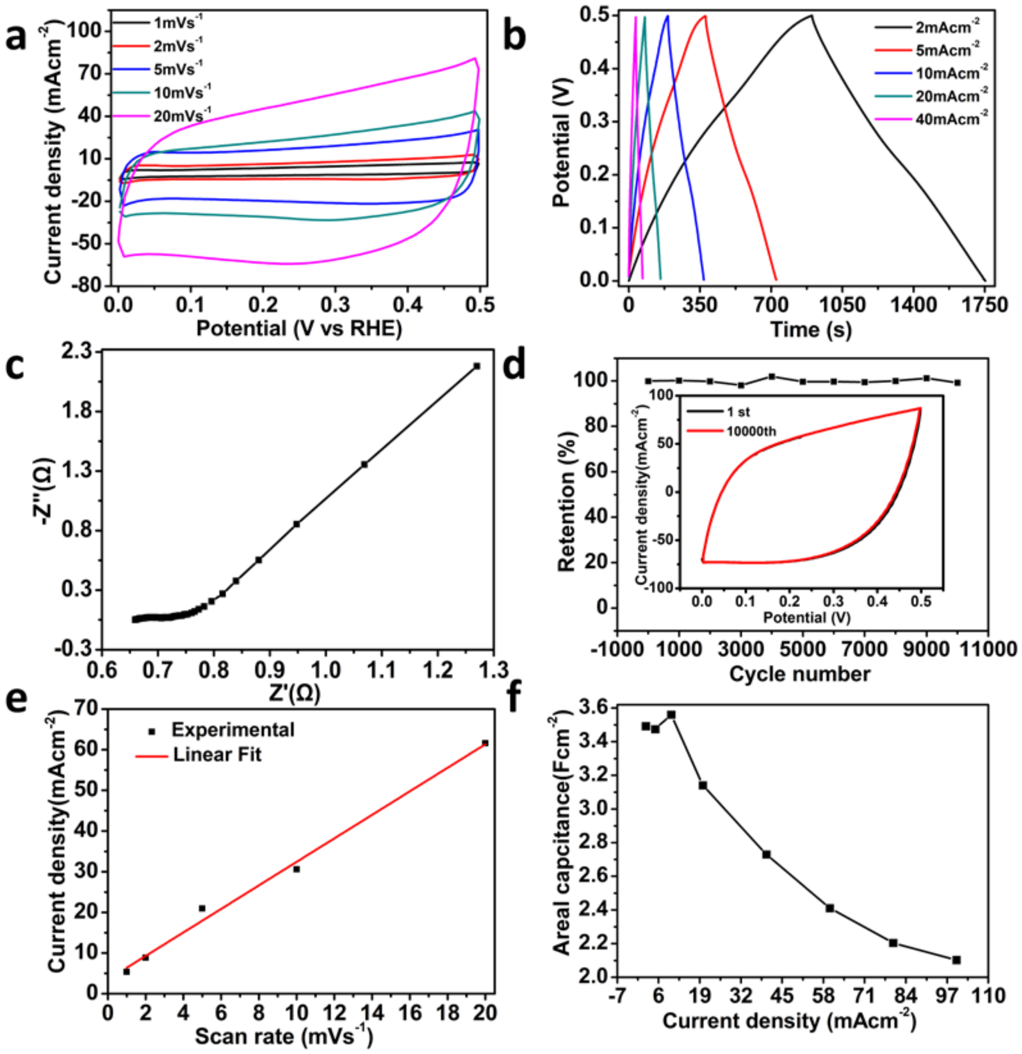

Supplementary Fig.13. Electrochemical performance of MoN single crystal in 0.5 M H_2SO_4 electrolyte. (**a**) CV curves at different scan rate of porous MoN single crystal. (**b**) GCD curves at different current densities of the MoN crystal. (**c**) Nyquist plots of MoN crystal electrode before cycling. (**d**) Cycling performance of MoN single crystal at a scan rate of 50 mV s^{-1}; inset shows the CV curves of the 1st and 10000th cycles. (**e**) Linear dependence of current density on scan rate of CVs at scan rates from 1 to 20 mV s^{-1}. (**f**) Areal capacitance as a function of current densities of MoN single crystal.

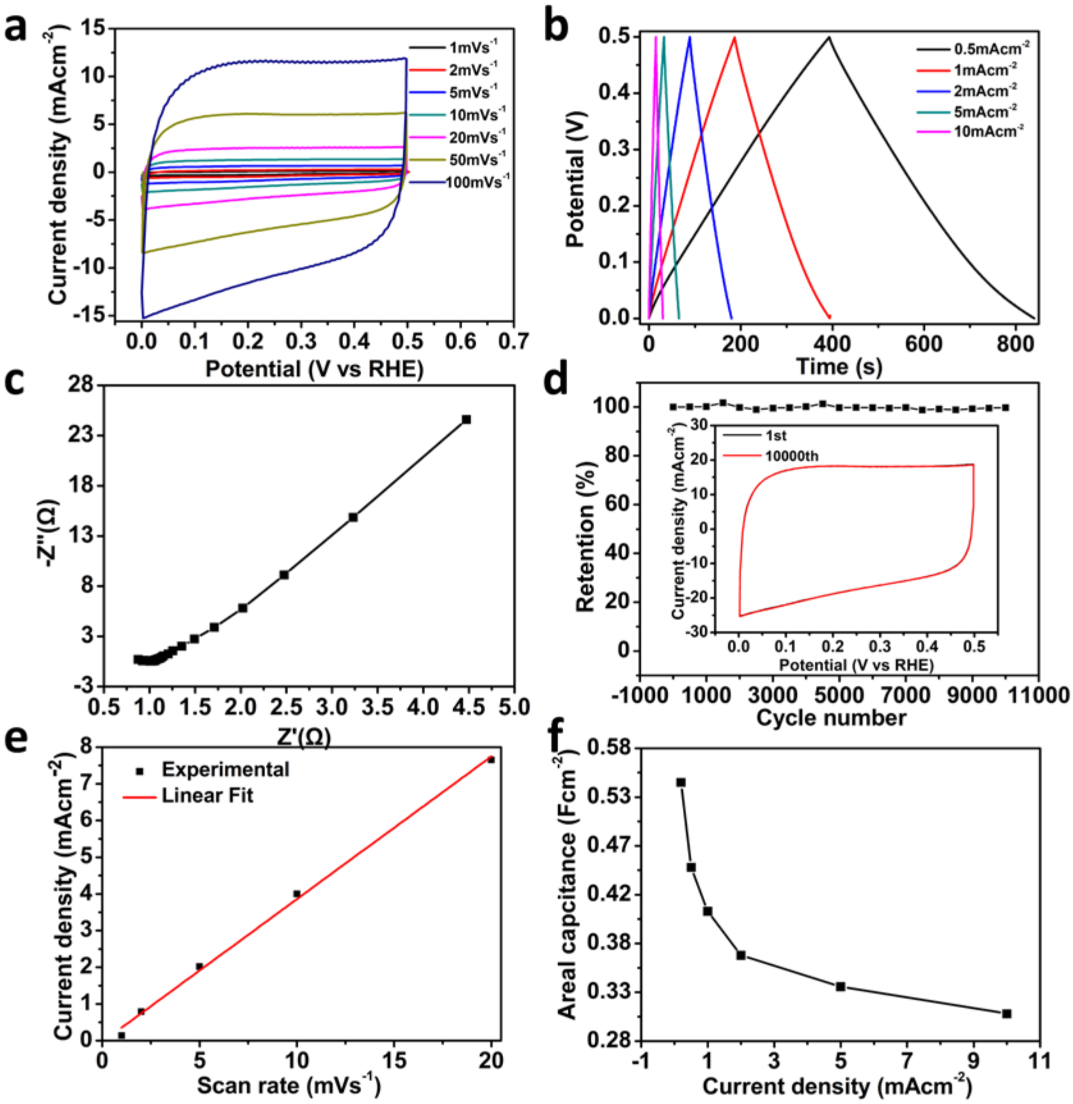

Supplementary Fig.14. Electrochemical performance of Ta_5N_6 single crystal in 0.5 M H_2SO_4 electrolyte. (**a**) CV curves at different scan rate of porous Ta_5N_6 single crystal. (**b**) GCD curves at different current densities of the Ta_5N_6 crystal. (**c**) Nyquist plots of Ta_5N_6 crystal electrode before cycling. (**d**) Cycling performance of Ta_5N_6 single crystal at a scan rate of 50 mV s^{-1}; inset shows the CV curves of the 1st and 10000th cycles. (**e**) Linear dependence of current density on scan rate of CVs at scan rates from 1 to 20 mV s^{-1}. (**f**) Areal capacitance as a function of current densities of Ta_5N_6 single crystal.

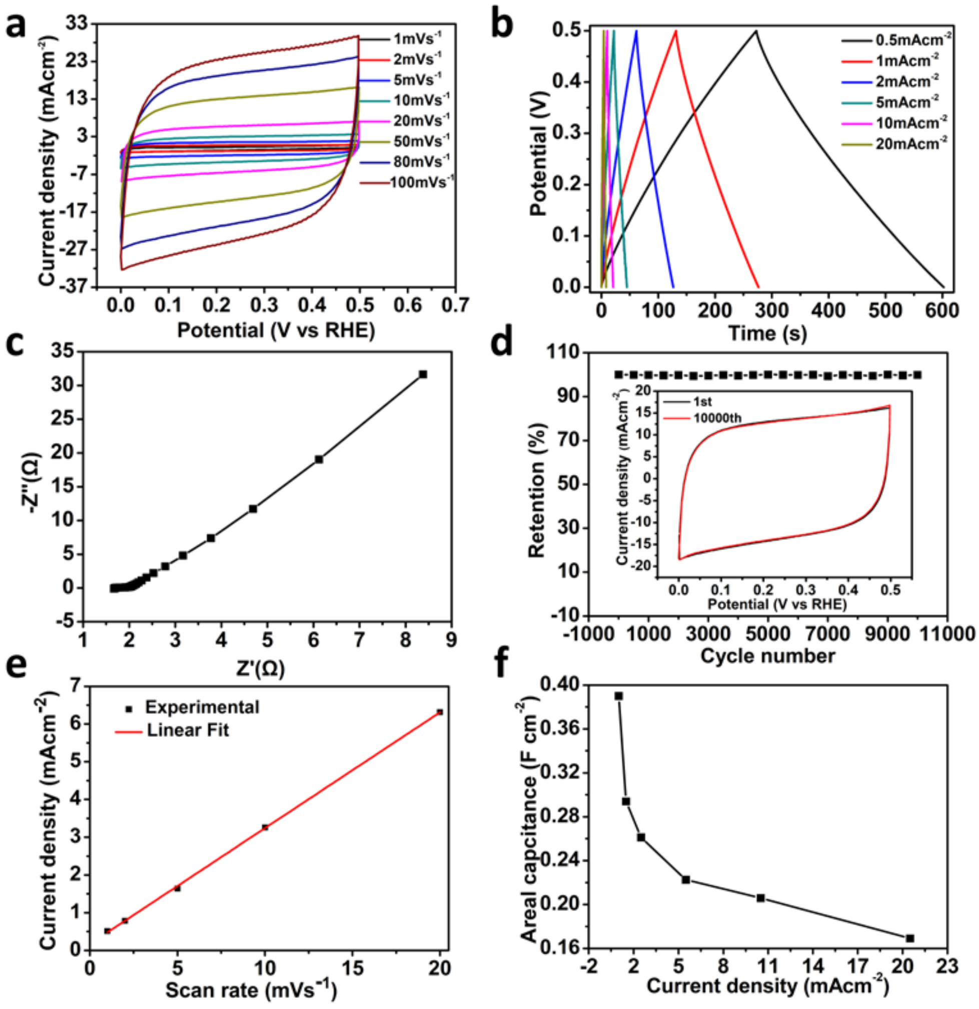

Supplementary Fig.15. Electrochemical performance of Ta_5N_6 single crystal in 1 M KOH electrolyte. (**a**) CV curves at different scan rate of porous Ta_5N_6 single crystal. (**b**) GCD curves at different current densities of the Ta_5N_6 crystal. (**c**) Nyquist plots of Ta_5N_6 crystal electrode before cycling. (**d**) Cycling performance of Ta_5N_6 single crystal at a scan rate of 50 mV s^{-1}; inset shows the CV curves of the 1st and 10000th cycles. (**e**) Linear dependence of current density on scan rate of CVs at scan rates from 1 to 20 mV s^{-1}. (**f**) Areal capacitance as a function of current densities of Ta_5N_6 single crystal.

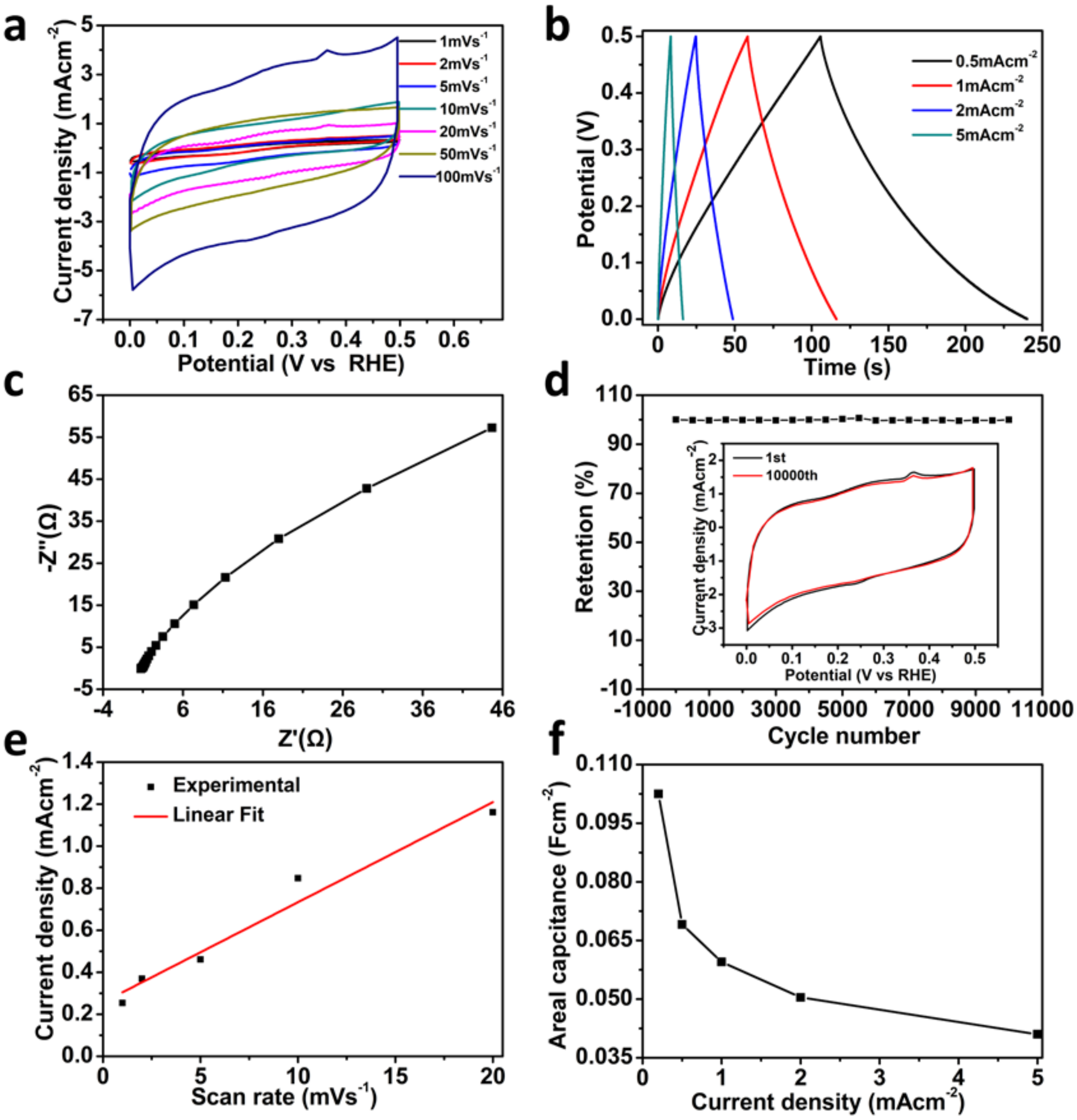

Supplementary Fig.16. Electrochemical performance of TiN single crystal in 0.5 M H_2SO_4 electrolyte. (**a**) CV curves at different scan rate of porous TiN single crystal. (**b**) GCD curves at different current densities of the TiN crystal. (**c**) Nyquist plots of TiN crystal electrode before cycling. (**d**) Cycling performance of TiN single crystal at a scan rate of 50 mV s^{-1}; inset shows the CV curves of the 1st and 10000th cycles. (**e**) Linear dependence of current density on scan rate of CVs at scan rates from 1 to 20 mV s^{-1}. (**f**) Areal capacitance as a function of current densities of TiN single crystal.

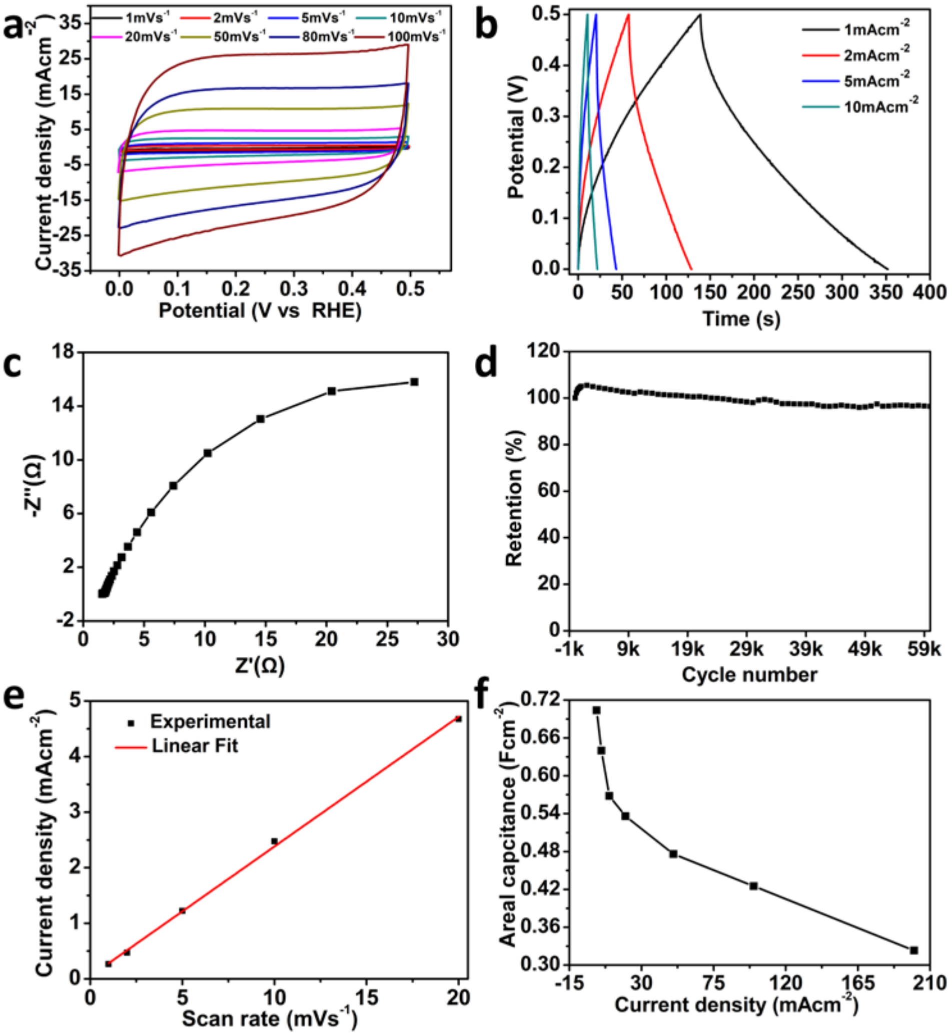

Supplementary Fig.17. Electrochemical performance of TiN single crystal in 1 M KOH electrolyte. (**a**) CV curves at different scan rate of porous TiN single crystal. (**b**) GCD curves at different current densities of the TiN crystal. (**c**) Nyquist plots and of TiN crystal electrode before cycling. (**d**) Cycling performance of TiN single crystal at a scan rate of 50 mV s^{-1}. (**e**) Linear dependence of current density on scan rate of CVs at scan rates from 1 to 20 mV s^{-1}. (**f**) Areal capacitance as a function of current densities of TiN single crystal.

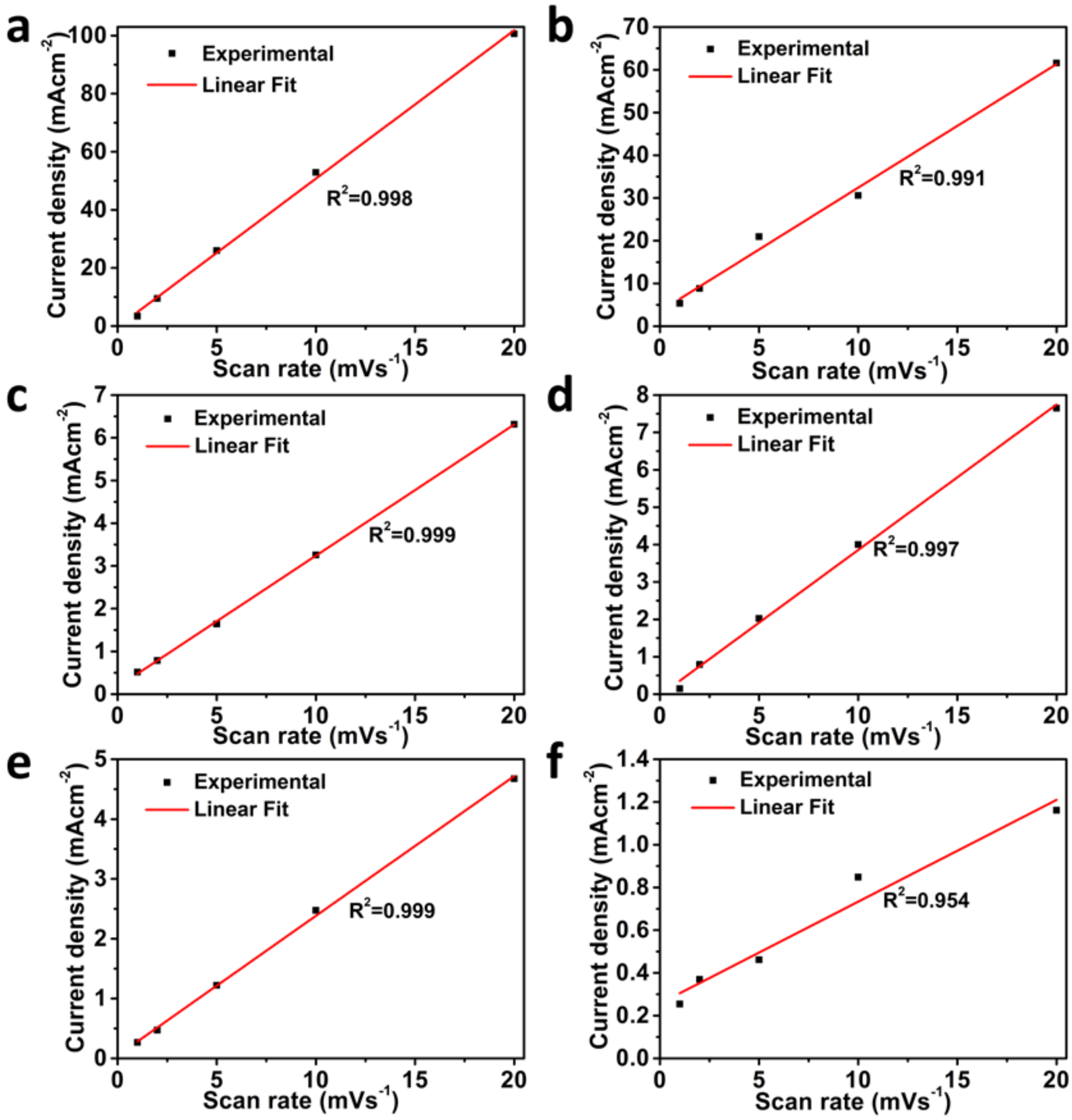

Supplementary Fig.18. **The linear fitting of dependence of current density on scan rate of CVs at 0.35 V.** (**a**) MoN in KOH. (**b**) MoN in H_2SO_4. (**c**) Ta_5N_6 in KOH. (**d**) Ta_5N_6 in H_2SO_4. (**e**) TiN in KOH. (**f**) TiN in H_2SO_4.

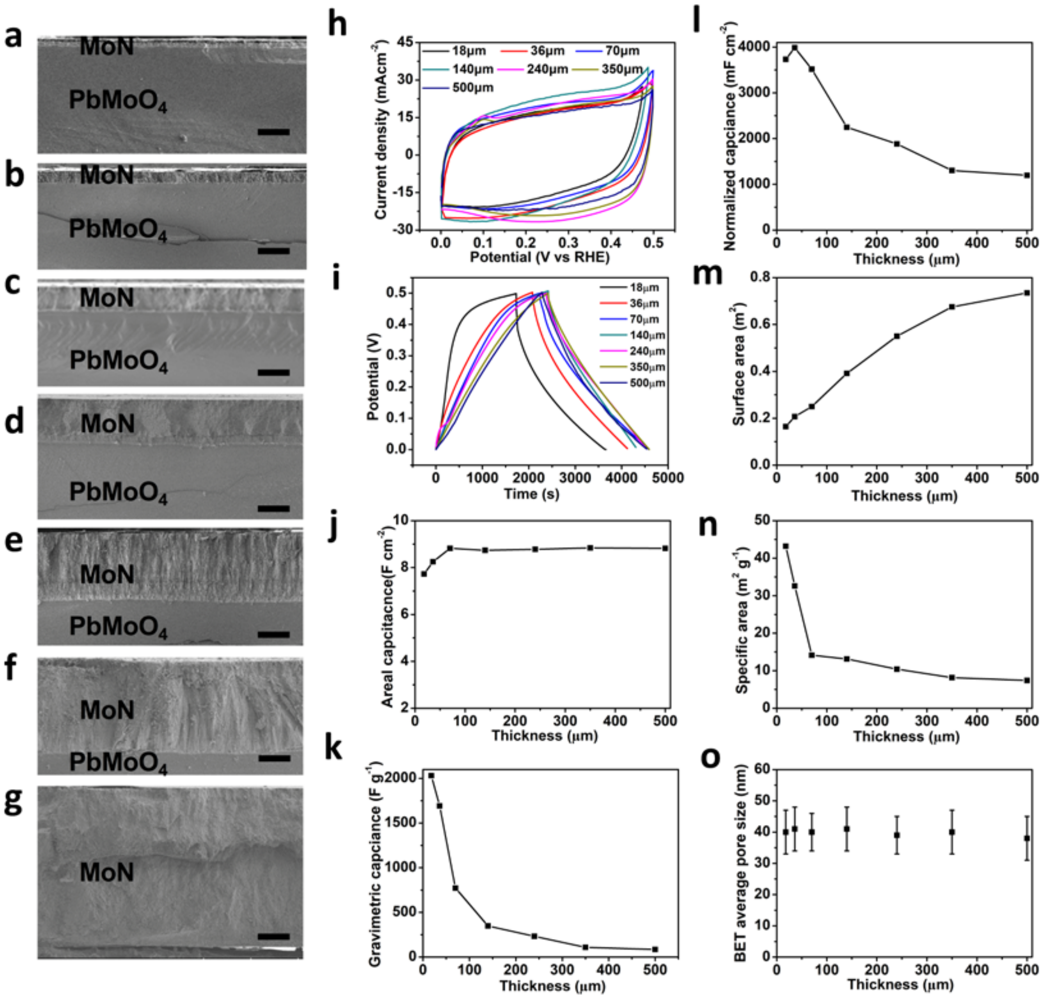

Supplementary Fig.19. Electrochemical performance of MoN single crystals with different thickness. (**a-g**) SEM image for the porous MoN single crystals with different thickness grown on the $PbMoO_4$ substrates with the area of 1 × 1 cm^2. (**h**) CV curves of porous MoN single crystals with different thickness at scan rate of 5 mVs^{-1}. (**i**) GCD curves of porous MoN single crystals with different thickness at the current density of 2 mA cm^{-2}. (**j**) The dependence of areal capacitance on the thickness of porous MoN single crystals at the scan rate of 2 mVs^{-1}. (**k**) The dependence of specific gravimetric capacitance on the thickness of porous MoN single crystals. (**l**) The dependence of normalized BET areal capacitance on the thickness of porous MoN single crystals. (**m**) BET surface areas of 1 × 1 cm^2 single crystals with different thickness grown on the $PbMoO_4$ substrates. (**n**) BET specific areas of the porous MoN single crystals with different thickness grown on the $PbMoO_4$ substrates. (**o**) The dependence of average pore size with different thick thickness of porous MoN single crystals. The scale bar is 100 μm in (**a-g**).

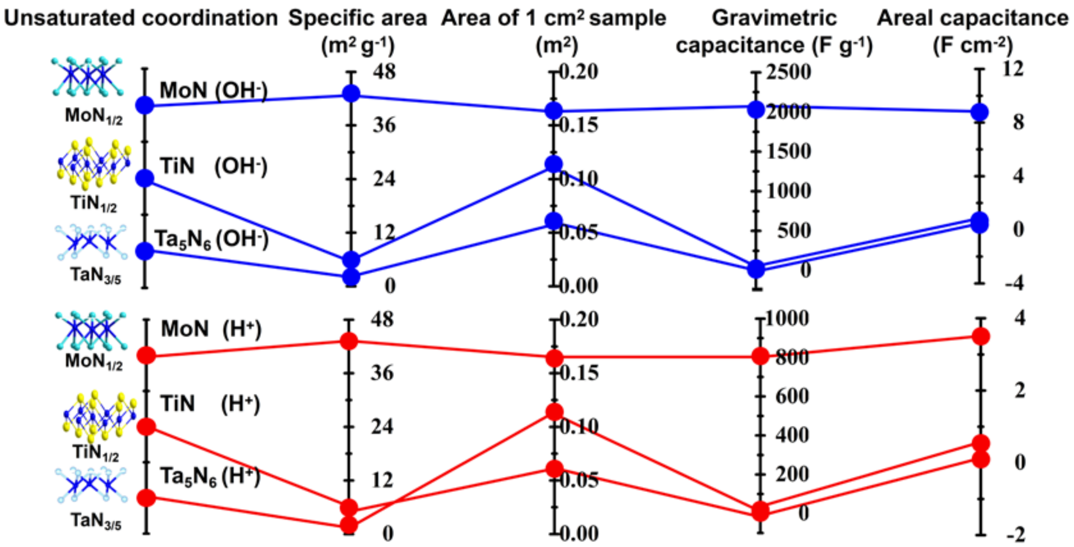

Supplementary Fig.20. **The relationship between active sites and performance.** The correlation between the unsaturated coordination structure, specific area, area of sample, gravimetric capacitance and areal capacitance for porous MoN, TiN and Ta_5N_6 single crystals.

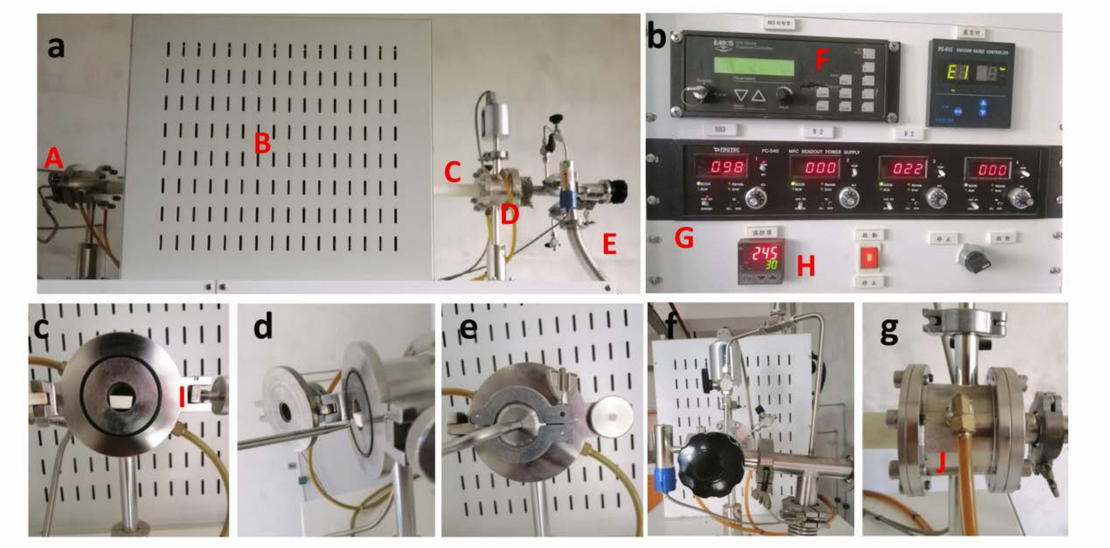

Supplementary Fig.21. **The digital photo of our vacuum system.** (**a**) Vacuum system. (**b**) Control panel. (**c**) The chamber for preparing sample. (**d**) Pushing samples into the chamber. (**e**) Sealing the chamber for preparing the sample. (**f**) The outlet tip of the system. (**g**) The steel cooling chamber of the vacuum system.

Supplementary Tables

Supplementary Table 1. Lattice parameter of the crystals. The comparison of calculated parameters, theoretical parameters and experimental values of of MoN, TiN and Ta_5N_6 while the theoretical and experimental values are obtained from references as listed below.

	Space group	Computed values (Å)	Theoretical Value (Å)	Experiment (Å)
MoN	P63/MMC	a=b=5.73, c=5.61	a=5.71, c=5.63 [23]	a=5.72 c=5.60 [24]
TiN	FM-3M	a=b=c=4.244	a=b=c=4.254 [25]	a=b=c=4.240 [26]
Ta_5N_6	P63/MMC	a=b=5.176, c=10.35	a=b=5.22, c=10.46 [27]	a=b=5.176 c=10.35 [28]

Supplementary Table 2. **The adsorption energies of H and OH on different surfaces.** The data are collected from references and summarized as listed in the table.

Surface	Energy (eV)	Surface	Energy (eV)
Ta_3N_5(100)-OH [29]	-2.3 ~ -3.3	MoC(111)-H [32]	-2.90 ~ -3.26
Ta(001)-H [30]	0.70 ~ -0.44	Mo(110)-OH [33]	-0.61
Ta(110)-H [31]	-2.35 ~ -3.27	Mo_2N(100)-H [34]	-2.74 ~ -3.14
Ta(100)-H [31]	-2.31 ~ -2.93	TiN-2(OH) [35]	-1.18 ~ -7.66
Mo(110)-H [31]	-1.56 ~ -3.08	TiO_2(001)-H [36]	-0.13 ~ -1.56
Mo(100)-H [31]	-1.89 ~ -2.99	TiO_2(100)-H [36]	-0.03 ~ -2.68

Supplementary Table 3. **The areal capacitance, stability and voltage window of reported materials.** The data is collected from the references as listed below.

Materials	Areal capacitance	Current load or scan rate	Retention	Voltage window	Reference
Porous MoN single crystal	8.8 Fcm^{-2}	5 $mAcm^{-2}$	100% after 10000 cycles	0-0.5 V *vs* RHE	**This work**
Nb_4N_5@NC	0.226 Fcm^{-2}	0.5 mVs^{-1}	100% after 2000 cycles	0-1.0 V *vs* Ag/AgCl	38
MnO_2 nanorods	0.22 Fcm^{-2}	0.75 $mAcm^{-2}$	95.5% after 5000 cycles	0-1.8 V *vs* SCE	39
HG-Ti_3C_2	4 Fcm^{-2}	5 mVs^{-1}	90% after 10000 cycles	-1.1-0.2 V *vs* SCE	40
Co_3O_4@MnO_2 nanowire array	0.7 Fcm^{-2}	4 $mAcm^{-2}$	97.3% after 5000 cycles	-0.2-0.6 V *vs* Ag/AgCl	41
Mesoporous $NiCo_2O_4$ nanosheets	3.51 Fcm^{-2}	1.8 $mAcm^{-2}$	93.3% after 3000 cycles	-0.2-0.6 V *vs* SCE	42
Carbon fiber cloth /MnO_2/CNTs	3.416 Fcm^{-2}	2 mVs^{-1}	100% after 1500 cycles	0-1.0 V	43
WO_{3-x}/MoO_{3-x} Core/Shell nanowires	0.216 Fcm^{-2}	2 $mAcm^{-2}$	75% after 10000 cycles	0-1.9 V *vs* RHE	44
Activated carbon fiber	1.56 Fcm^{-2}	5 $mAcm^{-2}$	100% 20000 cycles	0-1.0 V *vs* SCE	45
Ti-Doped Fe_2O_3@PEDOT	1.15 Fcm^{-2}	1 $mAcm^{-2}$	85.4% after 600 cycles	0-1.6 V *vs* SCE	46
PANI/Au/paper	0.8 Fcm^{-2}	1 $mAcm^{-2}$	100% after 10000 cycles	0-0.8 V	47
Hierarchical urchin-like Ni_3S_2	21.54 Fcm^{-2}	2 $mAcm^{-2}$	59.2% after 1000 cycles	0-0.6 V	48
$NiCo_2S_4$ nanotube arrays on Ni foam	14.39 Fcm^{-2}	5 $mAcm^{-2}$	92% after 5000 cycles	0-0.6V *vs* Hg/HgO	49
4-mm-thick Ultrahigh Loading MnO_2	44.13 Fcm^{-2}	0.5 $mAcm^{-2}$	92.9% After 20000cycles	0-0.8V vs SCE	50
